# Drug repurposing of pyrimidine analogs as potent antiviral compounds against human enterovirus A71 infection with potential clinical applications

**DOI:** 10.1038/s41598-020-65152-4

**Published:** 2020-05-18

**Authors:** Jialei Sun, Thinesshwary Yogarajah, Regina Ching Hua Lee, Parveen Kaur, Masafumi Inoue, Yong Wah Tan, Justin Jang Hann Chu

**Affiliations:** 1 Laboratory of Molecular RNA Virology and Antiviral Strategies, Department of Microbiology and Immunology, Yong Loo Lin School of Medicine, National University Health System, National University of Singapore, MD4 Level 5, 5 Science Drive 2, Singapore, 117597 Singapore; 2 Institute of Molecular and Cell Biology, Agency for Science, Technology and Research (A*STAR), 61 Biopolis Drive, Proteos #06-05, 138673 Singapore, Singapore; 3 Experimental Therapeutics Centre, Agency for Science, Technology and Research, Singapore, Singapore

**Keywords:** Drug discovery, Microbiology

## Abstract

Enterovirus A71 (EV-A71) is one of the aetiological agents for the hand, foot and mouth disease (HFMD) in young children and a potential cause of neurological complications in afflicted patients. Since its discovery in 1969, there remains no approved antiviral for EV-A71 and other HFMD-causing enteroviruses. We set out to address the lack of therapeutics against EV-A71 by screening an FDA-approved drug library and found an enrichment of hits including pyrimidine antimetabolite, gemcitabine which showed 90.2% of inhibition on EV-A71 infection. Gemcitabine and other nucleoside analogs, LY2334737 and sofosbuvir inhibition of EV-A71 infection were disclosed using molecular and proteomic quantification, and *in vitro* and *in vivo* efficacy evaluation. Gemcitabine displayed a significant reduction of infectious EV-A71 titres by 2.5 logs PFU/mL and was shown to target the early stage of EV-A71 viral RNA and viral protein synthesis process especially via inhibition of the RNA dependent RNA polymerase. In addition, the drug combination study of gemcitabine’s synergistic effects with interferon-β at 1:1 and 1:2 ratio enhanced inhibition against EV-A71 replication. Since gemcitabine is known to metabolize rapidly *in vivo*, other nucleoside analogs, LY2334737 and sofosbuvir conferred protection in mice against lethal EV-A71 challenge by potentially reducing the death rate, viral titers as well on virus-induced pathology in the limb muscle tissue of mice. Additionally, we found that gemcitabine is competent to inhibit other positive-sense RNA viruses of the *Flaviviridae* and *Togaviridae* family. Overall, these drugs provide new insights into targeting viral factors as a broad-spectrum antiviral strategy with potential therapeutic value for future development and are worthy of potential clinical application.

## Introduction

Enterovirus A71 (EV-A71) is one of the aetiological agents of hand, foot and mouth disease (HFMD), a highly infectious and common affliction of young children in the Asia Pacific Region^1–5^. In addition to causing HFMD, EV-A71 has also been associated with the development of neurological complications in a subset of patients, a potentially fatal progression of HFMD^6^. Currently, there is no specific antiviral or a widely-approved vaccine for the treatment or protection against EV-A71. In addition, the vaccine production in China is insufficient to cater for other Asia markets.

Several drug candidates have been identified through high throughput screening of libraries of pharmacologically active compounds and natural products. The drug candidates were found to target either the main capsid protein VP1, the viral protease activities, or the viral RNA polymerase, with some demonstrating significant efficacy against EV-A71 *in vitro* and/or in animal models^7–9^. However, so far none of these compounds have reached the global market, either because they have failed to display a satisfactory safety profile or because their efficacy and safety profiles remain to be established in humans. The lack of therapeutic options presents a challenge for the public health sector in the management and limiting of transmission of the highly communicable disease. Hence, it is of interest to fuel research into the development of effective antivirals targeting the aetiologic agents of HFMD, especially EV-A71.

Drug repurposing has been gaining foothold in the research scape to hasten the development of new drugs for treatment, by identifying new uses for already-in-use drugs with clinical data available. Gemcitabine, also known as 2’, 2’-difluoro 2’deoxycytidine or dFdC, is a pyrimidine antimetabolite and has been approved for the treatment of various types of cancer, such as pancreatic cancer and non-small cell lung cancer^10–12^. Gemcitabine can also inhibit the infection of several viruses, such as hepatitis C virus (HCV), human immunodeficiency virus (HIV) and influenza A virus (IAV)^13–15^. Gemcitabine is known to inhibit cancer and various viral infections by terminating chain elongation during DNA/RNA synthesis, thereby interrupting DNA/RNA synthesis^16,17^. Specifically, gemcitabine is understood to possibly inhibit Enteroviruses such as EV-A71 and Coxsackievirus B3^18^ with involvement of pyrimidine inhibition-induced innate immune response^19^. However, conflicting that theory gemcitabine was also propagated as a 3Dpol inhibitor in enterovirus infections^20,21^. Nonetheless, gemcitabine has not been used as an antiviral treatment in an animal model to prove its concept and mechanism.

In this study, a high-throughput screen was performed with an FDA-approved drug library and one of the hits, gemcitabine was selected for further evaluation and characterization of its anti-viral mechanism. In addition, LY2334737, the prodrug of gemcitabine and a nucleotide analog, sofosbuvir were found to exhibit inhibitory activity against EV-A71 infection *in vivo*.

## Materials and Methods

### Cell lines, media and virus strains

Human rhabdomyosarcoma (RD) cells (ATCC, #CCL136)] were used for the culture and titration of EV-A71. Baby hamster kidney cells (BHK21) were used for the culture of chikungunya virus (CHIKV) and dengue virus (DENV). RD and BHK cells were cultured at 37 °C with 5% CO_2_. Dulbecco’s modified eagle’s medium (DMEM) supplemented with 10% fetal bovine serum (FBS) was used in the culturing of RD cells and Roswell Park Memorial Institute medium (RPMI-1640) supplemented with 10% FBS was used in the culturing of BHK cells. FBS was reduced to 2% during virus infection assays for both cell lines. Seeded cells were EV-A71-infected with MOI of 1 at 37**°**C for 1 hour (h) in a CO_2_ incubator to allow virus adsorption for all assay unless otherwise mentioned. All mentioned experiments were carried out in accordance with the rules and guidelines under biosafety level 2 (BSL2) in view that all viruses were classified as BSL2 during the experiment performed.

Clinical isolate EV-A71 strain 5865/sin/000009 (EV-A71, GenBank accession number: AF316321) was used for both *in vitro* and *in vivo* assays. Other viruses used for *in vitro* assays: poliovirus type 1 Sabin strain (PV Sabin 1, GenBank accession number: AY184219), Coxsackievirus A6 (CV-A6, GenBank accession number: KC866983), Coxsackievirus A16 strain G-10 (CV-A16, Genbank accession number: U05876.1), echovirus 7 strain Wallace (E-7, GenBank accession number: AF465516), chikungunya virus strain SGEHICHD122508 (CHIKV, GenBank accession number: FJ445502.2) and dengue virus serotype 2 strain New Guinea C (DENV, GenBank accession number: KM204118.1).

### Viral plaque assay

Quantification of virus titres from *in vitro* assays were obtained from RD cells (2×10^5^) infected with 100 μL of serially diluted EV-A71 samples for 1 h at 37**°**C. The virus was removed by washing twice with phosphate buffered saline (PBS) and overlaid with 1 mL of DMEM containing 2% FBS and 0.5% agarose. The plates were stained and fixed overnight with a 10% paraformaldehyde-1% crystal violet solution at 72 hours post-infection (hpi). The agarose was removed, and viral plaques counted.

### Immunofluorescence assay (IFA)

EV-A71-infected RD cells were fixed and permeabilized with warm 4% paraformaldehyde containing 0.01% Triton-X for 10 min at room temperature, washed three times with PBS and stained with primary antibody mouse anti-EV-A71 VP2 protein (1:1000 dilution, Millipore, #MAB979) for 1 h at 37**°**C. The primary and secondary antibodies used were and followed by. The cells were then washed three times with PBS followed by staining with secondary antibody anti-mouse (IgG) antibody conjugated with FITC or 594 (1:1000 dilution, Millipore, #AP308F) for 1 h at 37**°**C. Cells were then washed three times with PBS and the cell nucleus was stained with 4′,6-diamidine-2′-phenylindole dihydrochloride (DAPI) for 15 min prior to imaging.

### High-throughput drug screening

A 1175-compound library of Food and Drug Administration (FDA)-approved drugs (Selleckchem #Z71700) was used for the screening assay. All compounds were dissolved in Dimethyl sulfoxide (DMSO) and stored at −80 °C. RD cells were seeded in 384-well plates at a density of 5000 cells per well and incubated overnight prior to EV-A71 infection. Compounds from the FDA drug library were diluted in DMEM and added at a final concentration of 10 μM. Mock-infected cell were included in all assay. DMSO (0.1%) was used as a negative control. Cells were then incubated for 12 h before fixed and stained by IFA as described above.

### High content imaging

FITC staining was imaged to represent EV-A71- infected cells and DAPI staining was imaged to represent total cells. For each sample, four images were taken and the image positions in each well were standardized to minimize imaging errors. The stable version (2.2.0) of CellProfiler downloaded from the CellProfiler website (www.cellprofiler.org)^22^ and installed on a PC (64-bit Windows 7 operating system) was used for the data processing. The infection ratio was defined as number of EV-A71-infected positive cells / number of total cells. The relative infection ratio of each of sample was then calculated by normalizing to the DMSO treatment control which was defined as having a relative infection ratio of 100%. Samples with <50% relative infection ratio were selected as hits. All imaging was obtained with the aid of automated Operetta High content imager (PerkinElmer) at 20× magnification.

### Drug post-treatment assay

The antiviral effects of selected hits against EV-A71 in the drug screening were validated by drug post-treatment assay. Briefly, 1 × 10^5^ RD cells were seeded in 24-well plates and incubated overnight prior to EV-A71 adsorption for 1 hours at 37**°**C. Directly after adsorption, cells were washed with PBS and treated with the different compounds at a range of concentrations for up to 12 hpi. Cell culture supernatants were collected after 12 h incubation and titred by plaque assay. Cell viability of the drug-treated cells was measured by alamarBlue cytotoxicity assay (Thermo Fisher Scientific) as described in manufacturer’s protocol.

### Time-of-addition and time-of-removal drug assays

RD cells (1 × 10^5^) were seeded in 24-well plates and incubated overnight at 37**°**C in a CO_2_ incubator prior to EV-A71 infection. EV-A71-infected cells were washed twice with PBS and topped up with DMEM with 2% FBS. For drug addition, 1 μM gemcitabine (Sigma, #G6423) was added at different time points (0, 2, 4, 6, 8, 10 hpi). While for drug removal, 1 μM gemcitabine was added at 0 hpi and removed at different time points (0, 2, 4, 6, 8, 10 hpi) and replaced with DMEM with 2% FBS media containing 0.1% DMSO. The drug-treated cell culture supernatants were harvest at 12 hpi and EV-A71 titre was detected by viral plaque assay.

### **Synergistic effects of gemcitabine with interferon (IFN)-β**

RD cells (1 × 10^5^) were seeded onto 24-well plates and incubated overnight. Cell monolayers were pre-treated with various concentrations of IFN-β for 24 h before EV-A71 infection. The EV-A71-infected RD cells were then treated with gemcitabine at different concentrations. For the IFN-β -gemcitabine combinatorial assay, RD cells were EV-A71-infected before treated with both compounds at different concentrations. Treatment with either compound alone was used as control. At 12 hpi, culture supernatants were harvested and virus titre was quantified by viral plaque assay.

### Quantitative real-time PCR (qRT-PCR) analysis

Total RNA was extracted from the EV-A71-infected and gemcitabine-treated RD cells at 3 hpi and 6 hpi using RNeasy mini kit (Qiagen, #74106). DMSO (0.1%) was used as treatment control. The relative abundance of viral RNA was detected using MD90/MD91 primers and SYBR green qRT-PCR kit (Sigma, #QR0100). Human β-actin was used as a loading control. Primers used for qRT-PCR in this assay are listed in Table 1.

**Table 1:** Primers used for EV-A71 RNA quantification.

Name	Sequence
MD90	ATTGTCACCATAAGCAGCCA
MD91	CCTCCGGCCCCTGAATGCGGCTAAT
β-actin-F	AGAGCTACGAGCTGCCTGAC
β-actin-R	AGCACTGTGTTGGCGTACAG

### Western blotting

The EV-A71-infected and gemcitabine-treated RD cells were incubated for 8 h before total cellular proteins were extracted using M-PER mammalian protein extraction reagent (Thermo Fisher Scientific, #78501) containing protease inhibitors (Halt Protease and Phosphatase Inhibitor Cocktail, Thermo Fisher Scientific, #78440) and EDTA. Proteins were separated in 10% polyacrylamide gel and transferred to nitrocellulose membranes using the Bio-Rad semidry transfer system. Mouse anti-EV-A71 VP2 protein (Millipore, #MAB979) was used to detect EV-A71 VP0 and VP2 proteins, mouse anti-EV-A71 3D protein (GeneTex, #GTX630193) was used to detect EV-A71 3D and 3CD proteins and mouse anti-actin (Millipore, #MAB1501) was used as loading control. Goat anti-mouse IgG conjugated to HRP (Thermo Fisher Scientific, #31430) was used as the secondary antibody.

### Dual-Glo luciferase assay for EV-A71 bicistronic construct

EV-A71 bicistronic plasmid with EV-A71 IRES expressing renilla luciferase (RLuc) was used as a measurement for viral protein synthesis, whilst pCMV expressing cap-dependent translation of firefly luciferase (FLuc) served as translation control. This EV-A71 bicistronic plasmid (500 ng) was transfected onto 24-well plate seeded RD cells (1 × 10^5^) using the jet-PRIME transfection system (Polyplus-transfection) according to manufacturer’s instructions. At 12 h post-transfection, cells were treated with various concentrations of gemcitabine (Selleckchem, #S4227) for 12 h. DMSO (0.1%) was used as a negative treatment control and 0.25 mg/mL amantadine was used as a positive treatment control in this assay^23^. Luciferase activity was detected by Dual-Glo luciferase assay system (Promega, #E2920) using GloMax-Multi detection system. Ratio of FLuc:RLuc was calculated to determine EV-A71 IRES-dependent translation inhibition by gemcitabine.

### NanoLuc replicon assay

The P1 region of a full-length EV-A71^24^ infectious clone was substituted with the gene encoding for Nano-luciferase (Promega). To generate the replicon-deficient 3D mutant, site-directed mutagenesis was performed using In-Fusion HD cloning (Clontech) to remove 53 amino acids from the C-terminus of 3D. RNA transcripts of either clone was produced using MEGAscript T7 transcription kit (Life Technologies) and products were purified using RNeasy kit (Qiagen). The transcripts were verified for integrity and concentration using agarose-gel electrophoresis before transfection. 100 ng of purified RNA transcripts of each construct were reverse-transfected into 20,000 RD cells on white 96-well plates (Corning) using Dharmafect-1 (Thermo Fisher Scientific). At 4 h post transfection, compound-containing media was substituted into each well and the samples were incubated for a further 12 h before luciferase detection using the Nano-Glo kit from Promega.

### **Toxicity*in vivo***

Suckling BALB/c mice were used in this study. The body weight loss of drug-treated mice was used as a marker for cytotoxicity effects. The body weights of drug-treated mice were measured daily for 15 days, the initial average weight of suckling mice (5–day-old) were normalized to 100% and ratios of the average weight on each day to the initial average weight were calculated. LY2334737 (MedChem Express, #HY-13672) was given to 6–day-old suckling BALB/c mice by oral feeding with dose of 0.32 mg/kg per mouse. Sofosbuvir (Selleckchem, #S2794) was administered to 6–day-old suckling BALB/c mice by intraperitoneal (i.p.) injection with dose of 3.5 mg/kg per mouse. Naive 5 or 6 day-old suckling BALB/c mice were used as negative controls for cytotoxicity study.

### **Antiviral evaluation*in vivo***

Suckling BALB/c mice (6 days old) were infected with EV-A71 (2 × 10^7^ PFU per mouse) prior to drug inoculation adapted from our previous work^25^. Briefly, a total of 5 doses were given at every 24 h interval from 1 hpi to 4 days post-infection (dpi). The dose and administration route of drug used was: 0.25 mg/kg for gemcitabine, i.p and 2 mg/kg for sofosbuvir, i.p. While 0.32 mg/kg for LY2334737 by oral feeding (equimolar to 0.25 mg/kg gemcitabine) were used. Drugs were prepared in 50 µL of PBS and the same volume of PBS was used for treatment controls. Upon EV-A71 infection, the survival of mice was monitored with a scoring system in 4 categories. Activity: 0 normal, 1 lethargy/abnormal posture, 2 huddled/inactive, 3 moribund/seizure; Breathing: 0 normal, 1 rapid/shallow, 2 rapid abdominal, 3 blue; Movement: 0 normal, 1 weakness, incoordination, 2 single limb dragging/paralysis, 3 multiple limb dragging/paralysis; Body weight: 0 normal, 1 loss of 5% over 24 h, 2 loss of more than 15% or up to 10% in 24 h, 3 loss of more than 10% over 24 h or 20% in total. A total of 6 or more points accumulated across all categories was determined as a humane endpoint and mice with such a score were euthanized.

### Quantification of viral titer and immunohistochemistry in mouse tissues

EV-A71-infected mice were sacrificed on 7 dpi and mice tissues were collected into CK14 homogenizing tubes (Bertin Corp). The tissues were weighed, 1 mL of DMEM was added into the tubes before homogenization using an orbital shaker at 6000 × g for 10 s. The process was repeated 5 times. The homogenized tissues were centrifuged for 10 min at 8000 × g, 4 °C to pellet tissue debris. Supernatants were collected, and the viral load was titrated by viral plaque assay. For histopathology studies, hind limbs from the EV-A71-infected mice were harvested on 7 dpi, fixed in 4% paraformaldehyde (Sigma) dissolved in PBS for 1 week at 4 °C, decalcified by Decalcifier II solution (Leica, #3800420) and embedded. Tissue damage was evaluated by haematoxylin and eosin (H&E) staining and EV-A71 antigen was detected by immunohistochemistry staining (IHC) using commercially available anti-EV-A71 antibody (MAB979, 1:200 dilution) and image captured using the Leica Bond-Max system.

### Ethical Approval

The animal experiments were carried out in accordance with the rules and guidelines under animal biosafety level 2 (ABSL2) containment at Center for Life Science (CeLS) with approval of the Committee for Laboratory Animal Research (NACLAR) in National University of Singapore. The protocols were reviewed and approved by the Institutional Animal Care and Use Committee (IACUC) of National University of Singapore (reference number: 2014-00078).

### Statistical analysis

All experiments were performed with at least biological duplicates. Data were shown with error bars indicating the standard deviations. Paired t-test was performed for all the *in vitro* experiments as well as viral load quantitation from mice organs. Microsoft excel and Graphpad prism was used for all the statistical analyses and statistical significances were denoted with asterisks, indicating p-value of <0.05.

## Results

### High throughput screening of the FDA-approved library against EV-A71 infection

A high throughput screen against the FDA-approved drug library was performed at 10 mM with 0.1% DMSO as a negative control. Mock-infected controls determined the background signals detected to be between 5% and 8%, setting the lower threshold of positive signal. After normalization of wells treated with 0.1% DMSO as to 100% infected wells, we validate the screen results.

**Figure 1:**
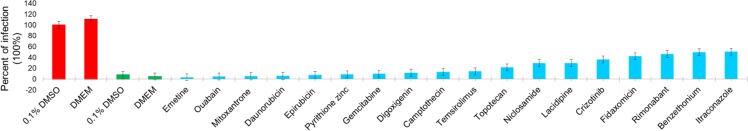
Hits of drug screening of an FDA-approved drug library. A high-throughput screening was performed for an FDA-approved drug library using immunofluorescence assay (IFA) on EV-A71-infected RD cells. All hits were compared with 0.1% DMSO control which was normalized to 100% infection. Cut off percentage for selected hits were determined at ≥50%. 18 hits were profound with inhibition against EV-A71. Infected controls are represented by red bars and mock-infected controls are represented by green bars.

Analysis of the results from the high throughput screen (Fig. 1) identified 18 hit compounds with ≥50% inhibition of infection (blue bars). Among the hits, emetine, the most effective compound, is known to inhibit protein synthesis by targeting 40 S subunit of the ribosome interfering with viral replication^26^. Three ion channel inhibitors were identified: ouabain, pyrithione zinc, and lacidipine. Ouabain is an inhibitor for Na + , K + −ATPase^27^. Pyrithione zinc is a proton pump inhibitor^28^ and lacidipine a calcium channel inhibitor^29^. We also found five inhibitors for topoisomerase I/II: mitoxantrone, daunorubicin, epirubicin, topotecan, and camptothecin which inhibits enteroviruses RNA replication and translation in a TOP1-dependent manner^30^. Two compounds with previously reported in cell signaling were also identified: temsirolimus and crizotinib. We found benzethonium which acts as an anti-microbial agent against rotavirus^31^. Itraconazole, another hit acts as a broad-spectrum inhibitor of enteroviruses, cardiovirus, and hepatitis C virus (HCV) by the inhibition of host proteins oxysterol binding protein (OSBP) and OSBP-related protein 4 (ORP4), both of which regulate cellular lipid-shuttling^32^. Other hits include fidaxomicin, digoxigenin, niclosamide, and rimonabant which are involved in various pathways, the functions of which are unclear to viral replication. Our interest focused on gemcitabine which showed 90.2% of inhibition on EV-A71 infection and was the top 7^th^ compound in the hit list. Gemcitabine has been shown to poses wide anti-viral function by interrupting DNA/RNA synthesis of several viruses^13–15,17^, however, their antiviral role in enterovirus infection is still unclear.

### Gemcitabine is a broad-spectrum antiviral compound

**Figure 2:**
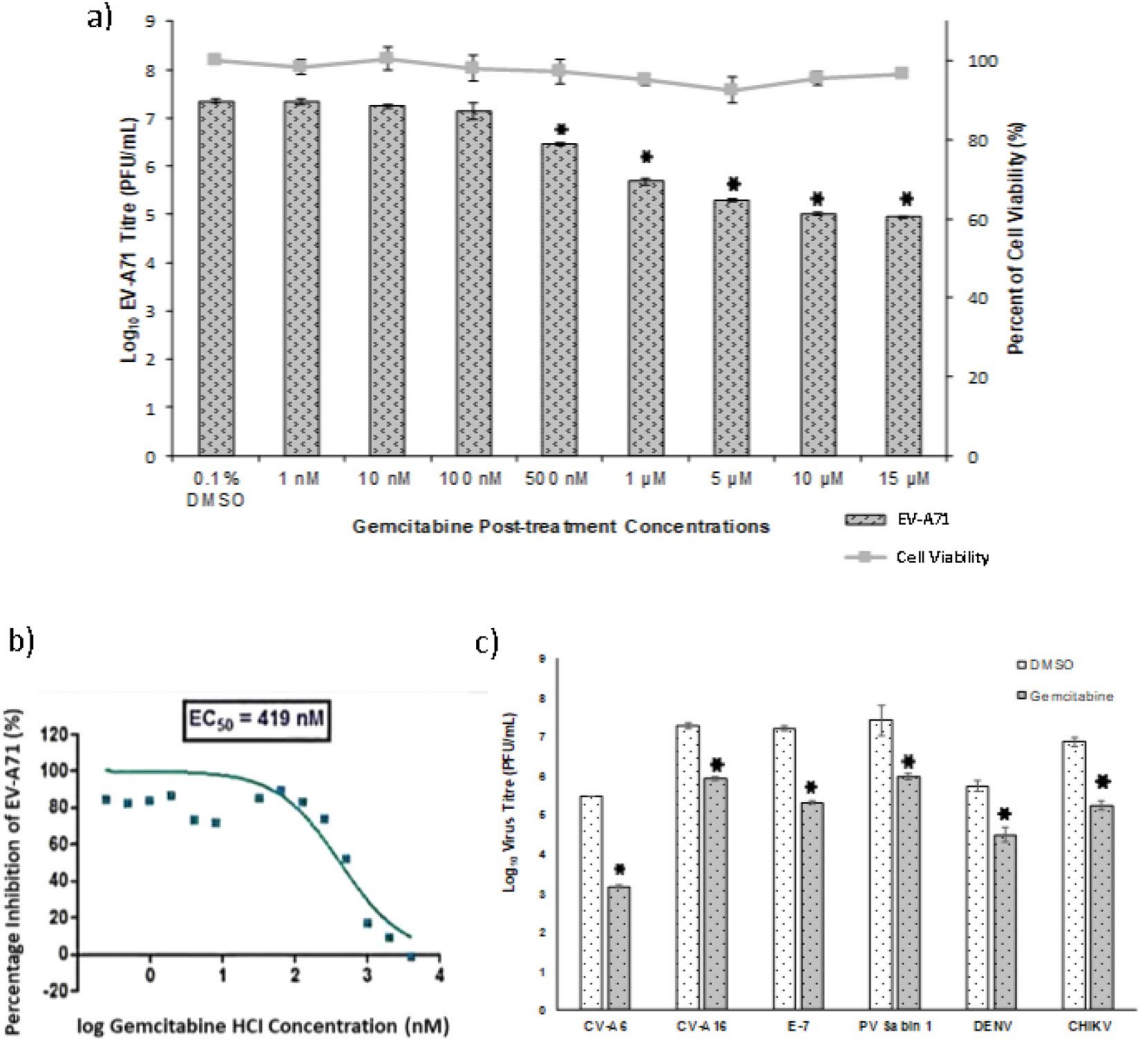
Inhibition of virus replication by gemcitabine. (**a**) RD cells were infected with EV-A71 at MOI. 1 and treated with gemcitabine at various concentrations (from 1 nM to 15 μM) to determine viral titer inhibition and cell viability. Concentration of ≥500 nM was able to inhibit EV-A71 infection. (**b**) Curve fitting using non-linear regression analysis was used to determine EC50. It was conditioned at 419 nM. (**c**) Gemcitabine inhibition on a broad range of viruses was determined using RD cells infected at MOI. 1 for enteroviruses and MOI.10 for DENV and CHIKV. Gemcitabine showed protection for all selected virus infection. The total virus titer was quantified by viral plaque assay. *p < 0.05, paired *t-test*, compared to DMSO control.

To validate the inhibitory effect of gemcitabine on EV-A71 infection, virus-infected RD cells were treated with gemcitabine at various concentrations (from 1 nM to 15 μM) and compared with treatment control, 0.1% DMSO (Fig. 2a). Gemcitabine significantly decreased the titre of EV-A71 between 1 to 2.3 log PFU/mL in a dosage-dependent manner with minimal cytotoxicity observed for all tested concentrations. Significant inhibition was observed from 500 nM concentrations of gemcitabine onwards. The concentration with 50% inhibition of EV-A71 infection (EC50) was determined, by curve fitting using non-linear regression analysis, to be 419 nM (Fig. 2b).

To assess the broad-spectrum potential of gemcitabine, the antiviral activity of gemcitabine was evaluated in cells infected with CV-A6, CV-A16, E-7, PV Sabin 1, DENV and CHIKV. As shown in Figs. 2c, 1 μM gemcitabine treatment decreased virus titer by 2.31 log PFU/mL for CV-A6, 1.34 log PFU/mL for CV-A16, 1.90 log PFU/mL for E-7 and 1.44 log PFU/mL for PV Sabin 1. For non- human enteroviruses, 1.23 log PFU/mL and 1.64 log PFU/mL of virus titer reductions were observed for DENV and CHIKV, respectively. Taken together, the result demonstrated the broad-spectrum antiviral activity of gemcitabine across three genera of positive-sense RNA viruses.

### Gemcitabine inhibits viral RNA and protein synthesis during EV-A71 infection

**Figure 3:**
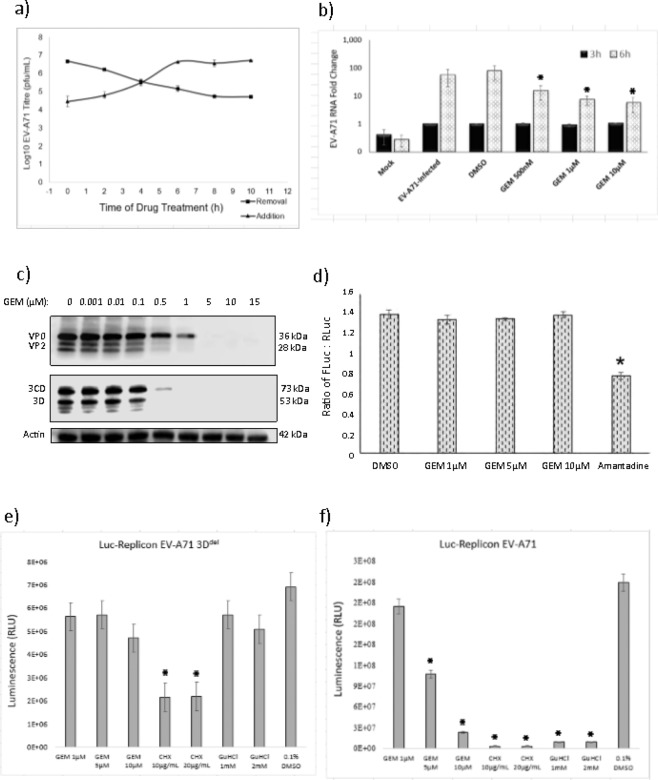
Inhibition of viral genomic RNA and protein synthesis by gemcitabine. (**a**) For time-of-drug-addition study, EV-A71-infected RD cells were treated with 1 μM gemcitabine at different time points. For time-of-drug-removal study, EV-A71-infected RD cells were treated with 1 μM gemcitabine and were removed at different time points. The EV-A71 inhibition curves crossed at 4 hpi showing early gemcitabine inhibition. (**b**) RD cells were infected with EV-A71 at MOI. 1 and treated with gemcitabine at three concentrations (0.5, 1 or 10 μM). The relative viral genome RNA fold change showed a significant reduction in all three-gemcitabine concentrations at 6 hpi. Gemcitabine is represented by Gem. (**c**) EV-A71-infected RD cells and treated with gemcitabine were assessed for viral protein synthesis by Western blotting. Gemcitabine was observed to inhibit non-structural (3CD and 3D) and structural protein (VP0 and VP2) synthesis from ≥0.5 μM gemcitabine. (**d**) FLuc: RLuc ratio was determined in RD cells transfected with EV-A71 bicistronic plasmid. Results showed a similar amount of FLuc: Rluc ratio for all gemcitabine concentration tested indicating no inhibition of IRES-dependent translation. (**e,f**) Luminescence readings from cells transfected with either replication-deficient replicon (3Ddel) or replication-competent construct. RLU showed a gradual decrease from 1 to 10 μM gemcitabine treated transfected cells specifically in the replication-competent construct. Average from three independent experiments are shown for each treatment with error bars representing standard deviation. The data were analyzed by one-way analysis of variance (ANOVA) followed by student’s T-test comparing against vehicle-control (0.1% DMSO) for each compound treatment and *indicates p < 0.05.

To gain a better understanding of the mechanism of action for gemcitabine in the inhibition of EV-A71 infection, time-of-addition and time-of-removal assays were performed. The viral replication process targeted by the drug can be identified by the crossing point of the graphs generated by the two assays, ie. an early crossing point of the two curves indicate that the drug inhibits an early infection process while a late crossing point indicates a late infection process being affected. In the time-of-addition assay (Fig. 3a), gemcitabine treatments earlier than 4 hpi were about 2 log units lower than that of 6 to 12 hpi in EV-A71 viral titer yields. For time-of-removal assay, compared with DMSO control (0 h removal) which had a titre of 6.67 log PFU/mL, gemcitabine treatment removed after 4 hpi had about 2 log units lower EV-A71 viral titre yield. This finding showed that the EV-A71 inhibition curves crossed at 4 hpi, suggesting an early event of EV-A71 infection inhibited by gemcitabine.

As gemcitabine is known to act as a chain terminator in DNA/RNA synthesis, its impact on viral RNA synthesis during EV-A71 infection was assessed using qRT-PCR. As shown in Fig. 3b, the amount of viral RNA increased between 3 and 6 hpi for all treated samples. Treatment with gemcitabine at 500 nM, 1 µM and 10 µM showed reduced EV-A71 genomic RNA fold changes of 15.33 ± 8.37, 7.35 ± 2.70 and 5.69 ± 3.19, respectively compared to DMSO control. The significantly lower viral titer in drug-treated cells may suggest that gemcitabine has an inhibitory effect on EV-A71 genomic RNA synthesis. The EV-A71 infected control (53.69 ± 32.38 fold change) and DMSO treated cells (78.03 ± 42.44 fold change) showed no experimental error and bias (Fig. 3b).

To further verify the inhibition effects of gemcitabine against EV-A71 infection, Western blotting was performed to assess viral protein expression. Compared to DMSO control, lower amounts of viral proteins, VP0, VP2, 3D and 3CD were observed in cells treated with concentrations of 500 nM gemcitabine and above (Fig. 3c). As the viral protein levels were assessed at 8 hpi, the low amount of protein production may reflect the lower viral RNA titers observed at 6 hpi (Fig. 3b). These results indicate that gemcitabine inhibits the synthesis of EV-A71 structural and non-structural proteins. To ascertain if viral protein synthesis initiation is inhibited by gemcitabine, a reporter assay to assess EV-A71 IRES-dependent translation was performed using the EV-A71 IRES bicistronic plasmid. RLuc was used as a measurement for viral protein synthesis, whilst FLuc served as translation control. Compared to the negative control (0.1% DMSO), minimal reduction of the FLuc: RLuc ratio was observed in 1, 5 and 10 µM gemcitabine-treated cells (Fig. 3d), suggesting that EV-A71 IRES-dependent translation was not affected by the treatment. Taken together gemcitabine may inhibit EV-A71 viral RNA synthesis, whilst a lesser extent of viral protein synthesis was affected as observed.

Gemcitabine’s effects on transcription and translation processes were understood using a previously generated DNA constructs of replication-competent or replication-incompetent EV-A71 that have their structural protein genes, VP1–4, replaced with the gene encoding for Nanoluciferase^30^. The replication-incompetent construct was constructed with a 53-amino acid deletion from the C-terminus of viral RNA dependent RNA polymerase 3D (3Ddel) that prevents RNA replication. This assay was performed using two positive control: a known inhibitor of enteroviral RNA replication, guanidine hydrochloride (GuHCl), and translation inhibitor, cycloheximide (CHX) (Fig. 3e,f). Hence in positive circumstances, RNA replication specific inhibitor will lower the expression levels of the luciferase gene in the replication-competent construct only. Gemcitabine showed a gradual decrease in nano-luciferase expression from 1 μM to 10 μM only in replication-competent construct transfected cells (Fig. 3f). Significantly inhibition in the accumulation of nano-luciferase in cells was achieved with treatment of 5 μM and 10 μM GEM (Fig. 3f), thus suggesting an inhibitory mechanism that may be directed against viral RNA replication.

### Synergistic anti-activity of gemcitabine in combination with IFN-β

**Figure 4:**
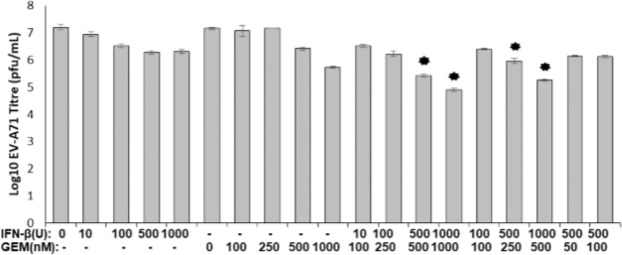
Combinational anti-EV-A71 effects of gemcitabine and IFN-b in infected RD cells. Synergistic effects of gemcitabine and IFN-β were well observed at ratio of 1:2 (250:500, 1000:500) and 1:1 (500 and 1000) with ~1 log reduction in viral titers. Data in this figure is represented as mean±standard deviation from three biological repeats. *p < 0.05, paired *t-test*, compared to DMSO control.

Drug synergy is performed by a combination of compounds to enhance drug efficacy making them therapeutically more specific. IFN-β, one of the natural host antiviral direct responses is commonly favored as a combinatory effect with drugs to boost antiviral effects^33–35^. Since IFN-β is usually upregulated during viral infection, synergistic use of this compound with drugs may benefit in the usage of lower doses of drugs to achieve efficient antiviral effects. Hence in this study, we evaluate the combinatorial effects of gemcitabine and IFN-β. As shown in Fig. 4, treatment with either IFN-β or gemcitabine showed a dose-dependent reduction of virus titre. Upon combination of gemcitabine and IFN-β at ratio of 1:2 (250:500, 1000:500) and 1:1 (500 and 1000), we found that virus titre reduction was significantly lower (~1 log reduction) than either IFN-β or gemcitabine treatment alone. These results suggest that IFN-β and gemcitabine may exert synergistic effects against EV-A71 infection.

### Sofosbuvir and LY2334737, but not gemcitabine, protect mice against lethal EV-A71 challenge

A significant limitation of gemcitabine use *in vivo* is its rapid metabolism by cytidine deaminase into its inactive uracil metabolite^36^. As such we used LY2334737, a gemcitabine prodrug generated by linking gemcitabine covalently to a valproic acid group, which prevents the binding to cytidine deaminase and inhibits subsequent deamination of gemcitabine^37^ improving its bioavailability *in vivo*. In light of the efficacy exhibited by gemcitabine against EV-A71, we also used an FDA approved nucleotide analog: sofosbuvir in *in vivo* treatment. It is a uridine analog already in use for the treatment of hepatitis C infections^38^. Similar to gemcitabine, sofosbuvir is a nucleotide analog and targets viral RNA polymerase and inhibits viral RNA synthesis^39^. Like LY2334737, sofosbuvir is covalently linked to protection chemical groups, phenoxyphosphorylamino propionic acid isopropyl ester, to prevent inactivation *in vivo*^38,40^. In addition, sofosbuvir contains a phosphate group that enhances the antiviral effect by promoting the phosphorylation of 2’-deoxy-2’-α-fluoro-β-C-methyluridine into its triphosphate have been proved in hepatitis C infection^41^.

**Figure 5:**
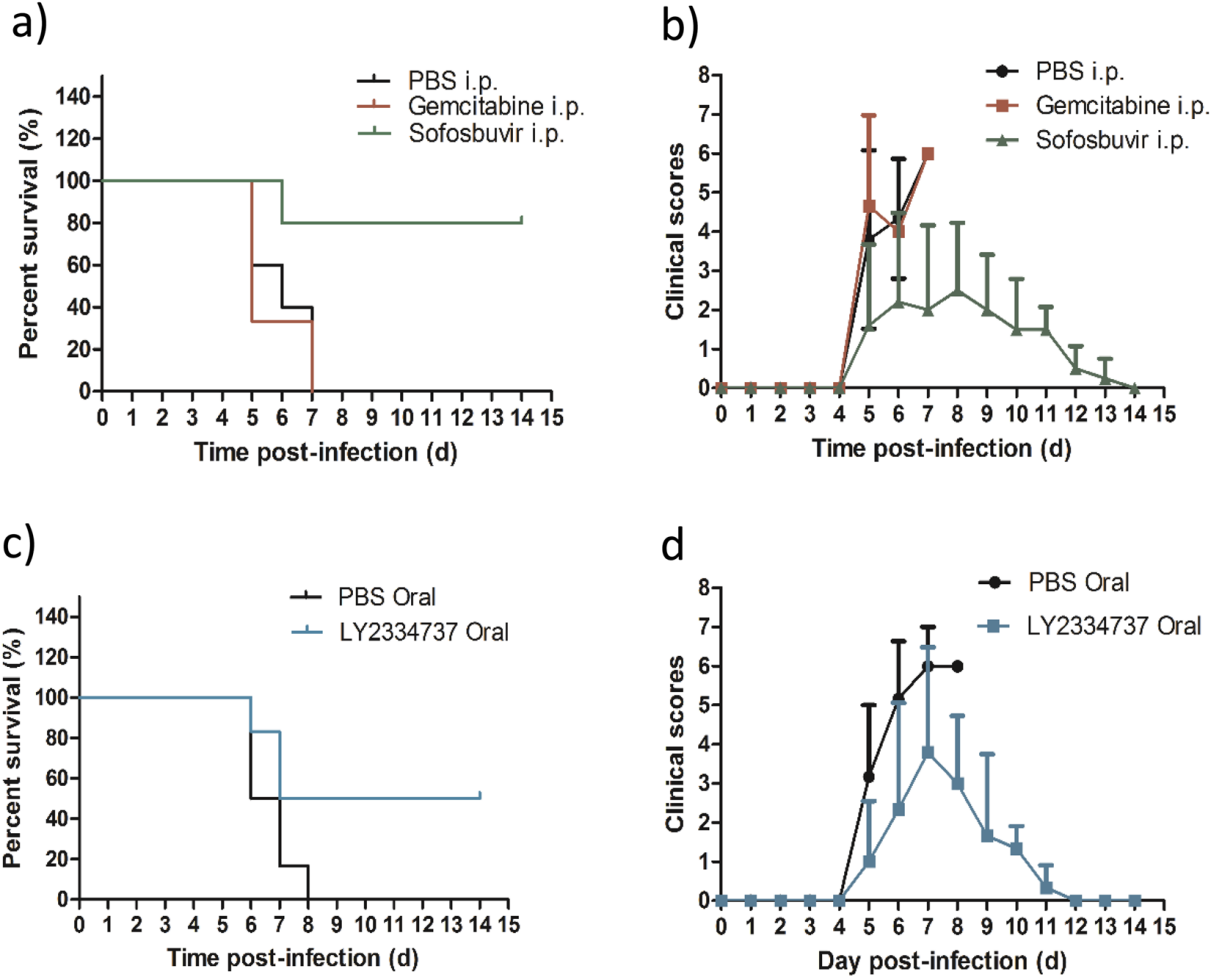
*In vivo* evaluation of gemcitabine, sofosbuvir, and LY2334737. 6-day-old BALB/c mice were infected with EV-A71 at a dose of 2 × 10^7^ PFU per mouse via intraperitoneal injection (i.p.). At 1 hpi, the EV-A71-infected mice were treated with first dose of drug. The dose of drug used was: 0.25 mg/kg for gemcitabine, 2 mg/kg for sofosbuvir and 0.32 mg/kg for LY2334737 (equimolar to 0.25 mg/kg gemcitabine). PBS was used as treatment control. The survival (*a* and *c*) and clinical scores (*b* and *d*) of the mice were recorded using the mice clinical scoring system for up to 14 dpi.

A 6-day-old BALB/c mice were injected intraperitoneally with a lethal dose of EV-A71 and treated with 5 doses of gemcitabine, or sofosbuvir. Mice injected with PBS were used as untreated control for gemcitabine and sofobuvir. As predicted possible due to rapidly metabolized by cystine deaminase, survival curves of the treatment groups showed no significant difference between the gemcitabine treated group and negative control PBS treated group (Fig. 5a). In contrast, sofosbuvir-treated mice showed improvement in survival rate of 80% up to 14 dpi (Fig. 5a). PBS control and gemcitabine-treatment showed severe clinical symptoms and high mean clinical scores (maximum of 6) with all the infected mice succumbed at 7 dpi (Fig. 5b). Contrary to PBS control, sofosbuvir-treated animals had presented with mild clinical signs and low mean of the clinical score (maximum of 3) with mice surviving up to 14 dpi (Fig. 5b). In view of gemcitabine known to be rapidly metabolized *in vivo*, it feasibly resulted in minimal protection against lethal challenge in mice^36^. However, sofosbuvir was deemed highly protective against EV-A71 infection *in vivo*.

To evaluate the antiviral efficacy of orally bioavailable LY2334737 against EV-A71 infection in mice, the drug was administered by oral gavage at 0.32 mg/kg and PBS was administered via the same route for control group. The LY2334737 treated group showed 50% of survival whilst PBS control exhibited 0% survival at 8 dpi (Fig. 5c). Mean clinical scores for LY2334737-treated group were lower (maximum of 4 with mild clinical signs) compared to the PBS control group which had high clinical score (maximum of 6 with severe clinical signs) (Fig. 5d). In conclusion, sofosbuvir and LY2334737, are efficacious against EV-A71 infections in the 6-days old murine model.

### Sofosbuvir, but not LY2334737 limits viral infection in the muscle tissue of EV-A71-infected mice

**Figure 6:**
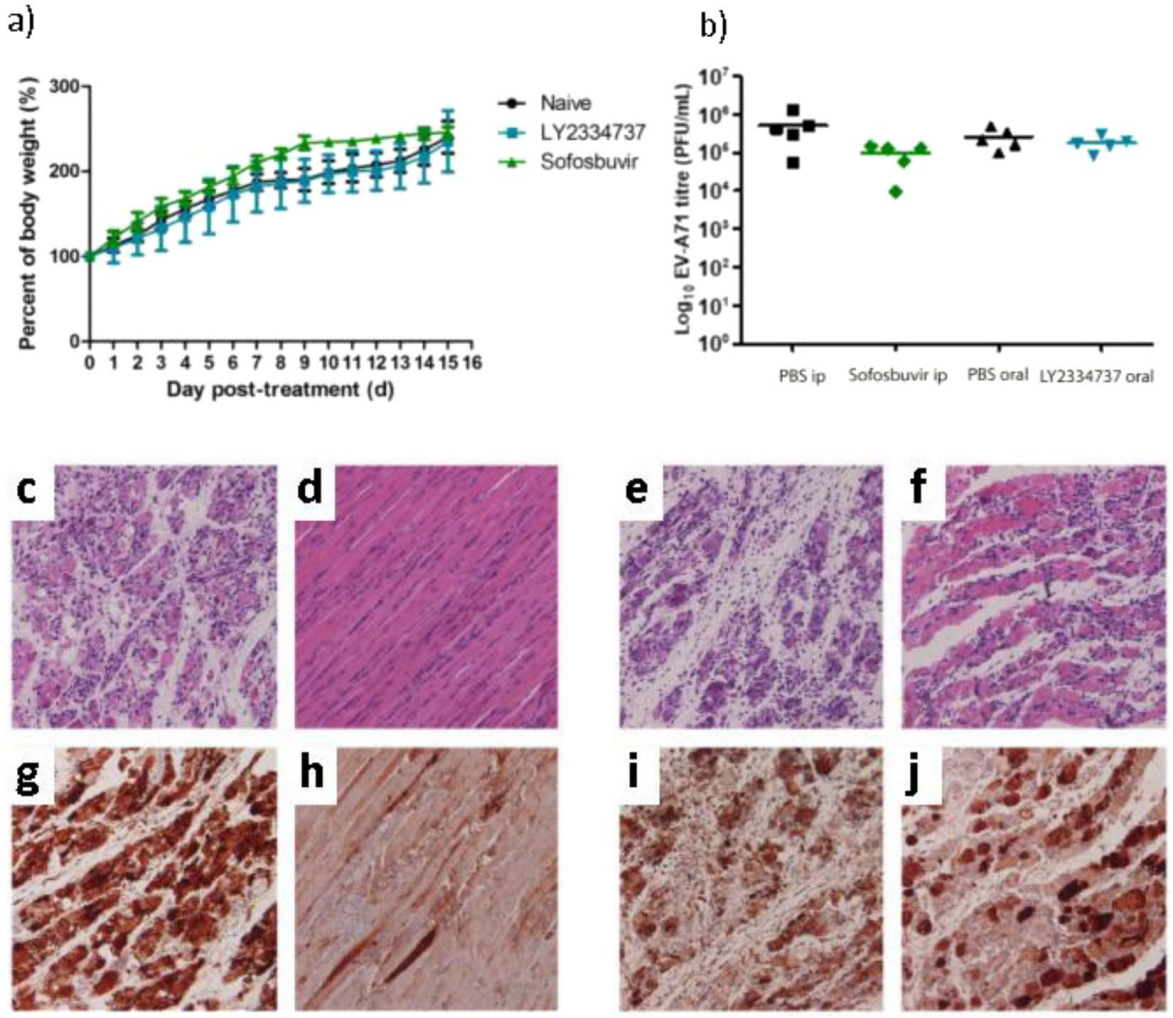
Viral load inhibition by LY2334737 and sofosbuvir on suckling BALB/c mice. (**a**) 6–day-old BALB/c mice were administered with 0.32 mg/kg LY2334737 or 2 mg/kg sofosbuvir at 0.32 mg/kg and the bodyweight of drug-treated mice was measured daily for 15 days. All mice survived treatment cytotoxicity challenge. Data in this figure is represented as mean±standard deviation. Viral load inhibition by sofosbuvir and LY2334737 in muscle tissue of EV-A71-infected mice were determined using plaque assay (**b**), H&E (**c–f**) and IHC (**g–j**). 6-day-old BALB/c mice were infected with EV-A71 at 2×10^7^ PFU per mouse via i.p. At 1 hpi, the EV-A71-infected mice were treated with first dose of drug. The dose of drug used was: 2 mg/kg for sofosbuvir and 0.32 mg/kg for LY2334737. PBS was used as treatment control. (**b**) A slight decrease in viral titer was observed in sofosbuvir treated mice compared to PBS control. Data in this figure was presented as mean and individual values. (**c–f**) H&E staining showed no obvious muscle necrosis in sofosbuvir-treated mice (**d**) and mild necrosis in LY2334737-treated mice (**f**), whilst PBS control showed extensive necrosis (**c,e**). (**g–j**) IHC staining muscle tissue showed extensive antigen positive in PBS control animals (**g,i**) while similar viral antigen distribution was observed in LY2334737 treated animals (**j**). However, low viral antigen is present in sofosbuvir treated animals (**h**). Data displayed are representative images of each group (n = 6). Magnification for H&E and IHC staining 20×.

To evaluate the *in vivo* cytotoxic effects of LY2334737 and sofosbuvir, 6–day-old BALB/c mice were administered with LY2334737 and sofosbuvir at 0.32 mg/kg and 2 mg/kg, respectively. The bodyweight of drug-treated mice was measured daily for 15 days. Non-treated naive mice were used as negative control for cytotoxic effects. As shown in Fig. 6a, LY2334737-treated mice showed similar body weight on each day post-treatment, as compared to the bodyweight of naive mice. Sofosbuvir showed a slightly higher body weight compared to naïve control. These results show that LY2334737 and sofosbuvir have minimal cytotoxic effects on suckling BALB/c mice.

To examine the impact of sofosbuvir and LY2334737 on viral infection and pathology in mice, muscle tissues of the EV-A71-infected mice from all treatment groups were harvested at 7 dpi and the viral titer were quantified by viral plaque assay. The results showed that while viral titer remains detectable in the limb muscle tissues upon drug treatment in all groups, a slight reduction of 0.7 log PFU/mL (p = 0.05) compared to PBS control (Fig. 6b) was noted for the sofosbuvir-treated group. In contrast, no significant change was detected in mice treated with LY2334737. Results from H&E staining performed on the limb muscle tissues revealed no obvious muscle necrosis in sofosbuvir-treated mice (Fig. 6d) and mild necrosis was noted for LY2334737-treated mice (Fig. 6f) compared to severe necrosis observed in PBS control (Fig. 6c,e). IHC staining muscle tissue showed low viral antigen presence in sofosbuvir treated animals (Fig. 6h), while similar viral antigen distribution was observed in LY2334737 treated animals (Fig. 6j) and PBS controls (Fig. 6g,i). These results demonstrated the efficacy of both sofosbuvir and LY2334737 in limiting EV-A71 infection and in particular sofosbuvir limits viral infection in the muscle tissue of EV-A71-infected mice.

## Discussion

Our study explored the possibility of identifying anti-virus drugs from the FDA-approved drug library. We showed that using high-throughput screening, we achieved to identify 18 hits, with emetine as the 1^st^ drug in the hit list. We chose to understand Gemcitabine inhibition using an animal model, as it has been shown to portray antiviral activity against enteroviruses, especially coxsackievirus B3, human rhinovirus and EV-A71^18,42,43^. Whereby, gemcitabine decreases proinflammatory cytokines, including TNF-α and IL-1β and, in an unknown manner, limits the UTP and CTP inhibiting human rhinovirus infection. Gemcitabine was also shown to target cellular Ribonucleotide Reductase Catalytic Subunit M interfering with Zika virus RNA transcription^42^. In our study, we showed that gemcitabine is a broad-spectrum antiviral compound against EV-A71, CV-A6, CV-A16, E-7, PV Sabin 1, DENV and CHIKV (Fig. 2). Our result using quantitative RT-PCR and western blot showed that gemcitabine inhibited the synthesis of viral RNA by targeting viral RNA dependent RNA polymerase (RdRp) during EV-A71 infection (Fig. 3). Gemcitabine also had synergistic effects with interferon-β and showed increase inhibition at selected concentrations of 1:2 and 1:1 ratio (Fig. 4). These results convinced the potent use of gemcitabine as an antiviral compound against a range of viruses. Furthermore, the synergistic effects of gemcitabine and IFN-β we not tested in animal models. Since both had different antiviral mechanisms as gemcitabine targets viral RNA synthesis while IFN-β inhibits viral infection by inducing the expression of interferon-stimulated genes (ISGs)^17,44^. As all type I IFNs have similar antiviral mechanisms, gemcitabine, in theory, would also have synergistic effects with other types I IFNs which can be explored. The combination of nucleotide analog inhibitors and protease inhibitors has been widely studied for HIV^45^ which indicates that the combination of these two types of inhibitors may present a promising strategy for antiviral discovery.

Nevertheless, *in vivo* findings showed that gemcitabine had minimal antiviral efficacy on EV-A71 (Fig. 5), as predicted due to the deamination of gemcitabine by cytidine deaminase^46^. Hence, usage of gemcitabine prodrug, LY2334737, offered protection against lethal EV-A71 challenge on mice, by improving survival rates up to 50%, while 2’-deoxy-2’-α-fluoro-β-C-methyluridine, sofosbuvir, as a potent inhibitor provided 80% protection on EV-A71-infected mice (Fig. 6). LY2334737 showed no significant difference in viral titer reduction, even though our result showed reduced muscle limb necrosis in mice. Possible LY2334737 as an oral prodrug composed of gemcitabine covalently linked to valproic acid by an amide bond at 4-(N)-position is released slowly from the gut into the systemic area as oppose to viral inoculation through the intraperitoneal infection^47^. Upon administration, LY2334737 is hydrolyzed by carboxylesterase 2 (CES2), releasing and activating gemcitabine^47,48^. The use of LY2334737 benefits from the unmodified gemcitabine as it allows the bypass of hydrolysis in enterocytes, eventually escaping the immense forefront metabolism^49,50^. The use of prodrug LY2334737 may benefit the shelf life of gemcitabine release since levels of LY2334737 are detectable several hours after oral administration. Considering that a gradual release of gemcitabine following cleavage of the amide bond and presence of valproic acid that limits cytidine deaminase deamination should enhance efficacy and systemic release of gemcitabine^50,51^. In addition, the prodrug strategy might also decrease the gastro-intestinal effects and immune-suppression effects caused by gemcitabine since LY2334737 does not degrade between pH 6 to 8 and slow hydrolysis in the liver and crude homogenates of small intestinal epithelial cells has been reported^47^. Apart from the limitation in this study of using i.p infection of virus, similar virus route of inoculation to LY2334737 may allow substantial antiviral effects to be observed. Furthermore, phase I trials of LY2334737 either as monotherapy or in combination with other agents are currently underway to determine the maximum tolerated dose and dose-limiting toxicities of daily administration particularly in cancer research^50^. Consequently, the administration of LY2334737 via oral route instead of injection or infusion makes it more convenient as an antiviral treatment and future optimization with similar dosage as clinical trials should be performed.

Sofosbuvir, on the other hand, is a nucleotide analog that can be metabolized into its active form 2’-deoxy-2’-α-fluoro-β-C-methyluridine-5’-triphosphate^52^. The fluoride present in sofosbuvir, as compared to gemcitabine, contributes to high electronegativity and low polarizability due to its multi-faceted chemical properties^52^. Hence, making this FDA approved RdRp inhibitor more stable for enzymatic cleavage compared to gemcitabine. Sofosbuvir is effective against a broad-range of viruses such as Dengue virus, hepatitis C, HIV and Zika virus^41,53–55^. It is widely used in human treatments against hepatitis C virus infection. Sofosbuvir in our i.p inoculated model showed promising results of protection against EV-A71 infection via reduced viral titer, reduced clinical scores based on symptoms and minimal muscle tissue necrosis (Fig. 6). Beneficially, our mice model was inoculated with 2 mg/kg per day of sofosbuvir as oppose to other Zika virus, CHIKV virus model and hepatitis C animal model which used ten times or more concentrated, 20 mg/kg to 80 mg/kg per day treatment via intraperitoneal infection^56–58^. Hence, the low usage of sofosbuvir achieved an 80% survival rate reflects sofosbuvir as an efficient inhibitor for EV-A71 as well as showed improvement in the reduction of the symptoms via clinical scoring. Sofosbuvir treated mice also showed better increase in weight contributing to the factor of reduction in ‘sick’ symptoms and an increase in health. Nevertheless, a previously reported Zika-infected mice model showed greater survival rate even when orally administered proving that various route of inoculation is possible with this compound and orally-treated EV-A71 can be tested to determine dosage of drug treatment^54^. Moreover, a variety of drug doses can be tested to determine the optimum dose suitable for potential clinical trials.

In conclusion, LY2334737 and sofosbuvir may be potential antivirals compounds against EV-A71 infection and could be tested against a wide range of viruses from *Picornaviridae* and *Flaviviridae* family. Hence, the effective use of these compounds against viruses could be further understood and proposed for clinical trials.

## Supplementary information

Supplementary information.
