# Supplementary material for: Drug repurposing of pyrimidine analogs as potent antiviral compounds against human enterovirus A71 infection with potential clinical applications
Source: Sci Rep. 2020 May 18;10:8159. doi: 10.1038/s41598-020-65152-4 (PMC7235037; doi:10.1038/s41598-020-65152-4)
Supplement: Supplementary file 1 — Supplementary information. [file 41598_2020_65152_MOESM1_ESM.docx]

**Drug repurposing of pyrimidine analogs as potent antiviral compounds against human enterovirus A71 infection with potential clinical applications**

Jialei Sun^1^, Thinesshwary Yogarajah^1^, Regina Ching Hua Lee ^1^, Parveen Kaur^1^, Masafumi Inoue ^3^, Yong Wah Tan ^2^, Justin Jang Hann Chu^1,2^

^1^ Laboratory of Molecular RNA Virology and Antiviral Strategies, Department of Microbiology and Immunology, Yong Loo Lin School of Medicine, National University Health System, National University of Singapore, MD4 Level 5, 5 Science Drive 2, Singapore 117597, Singapore.

^2^ Institute of Molecular and Cell Biology, Agency for Science, Technology and Research (A*STAR), 61 Biopolis Drive, Proteos #06-05, 138673, Singapore.

^3^ Experimental Therapeutics Centre, Agency for Science, Technology and Research, Singapore.

Author email address:

Sun Jialei (SJ) [sun_jialei@u.nus.edu](mailto:sun_jialei@u.nus.edu)

Thinesshwary Yogarajah (TY) [micthiy@nus.edu.sg](mailto:micthiy@nus.edu.sg)

Regina Lee Ching Hua (RLCH) [miclch@nus.edu.sg](mailto:miclch@nus.edu.sg)

Parveen Kaur (PK) [micpar@nus.edu.sg](mailto:micpar@nus.edu.sg)

Masafumi Inoue (MI) [minoue@eddc.a-star.edu.sg](mailto:minoue@eddc.a-star.edu.sg)

Yong Wah Tan [ywtan@imcb.a-star.edu.sg](mailto:ywtan@imcb.a-star.edu.sg)

Corresponding author:

Associate Professor Justin Jang Hann Chu

Laboratory of Molecular RNA Virology and Antiviral Strategies, Department of Microbiology and Immunology, Yong Loo Lin School of Medicine, National University Health System, National University of Singapore, MD4 Level 5, 5 Science Drive 2, Singapore 117597, Singapore

Email: [miccjh@nus.edu.sg](mailto:miccjh@nus.edu.sg)

**Supplementary Figure 1:** Full blot of detection of VP0 and VP2 protein of EV-A71.


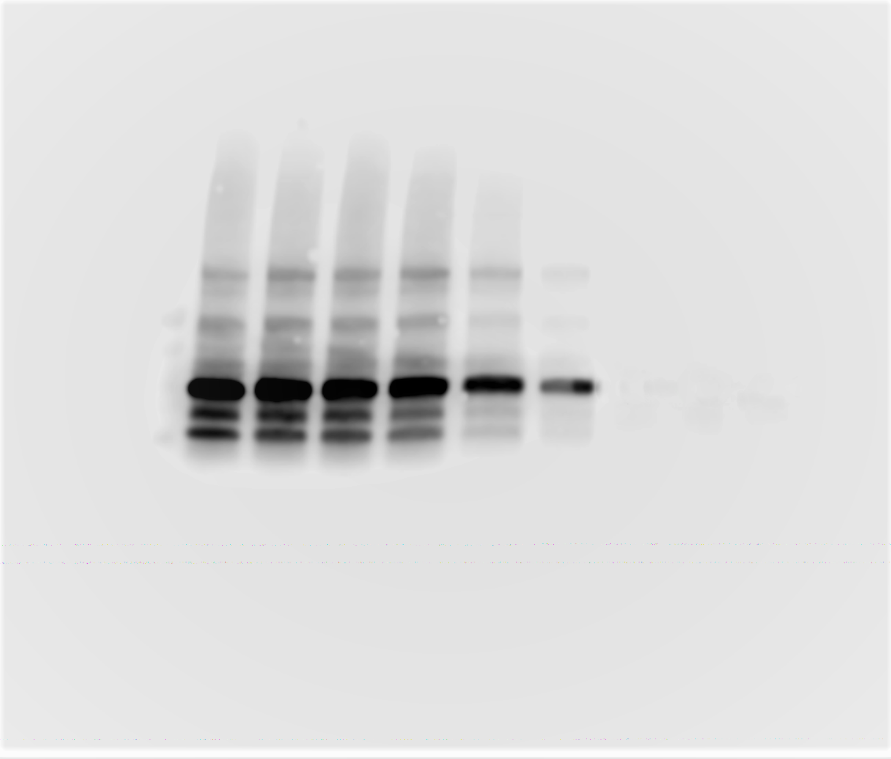


28 kDa

36 kDa

VP0

VP2

**Supplementary Figure 2:** Full blot of detection of 3CD and 3D protein of EV-A71.


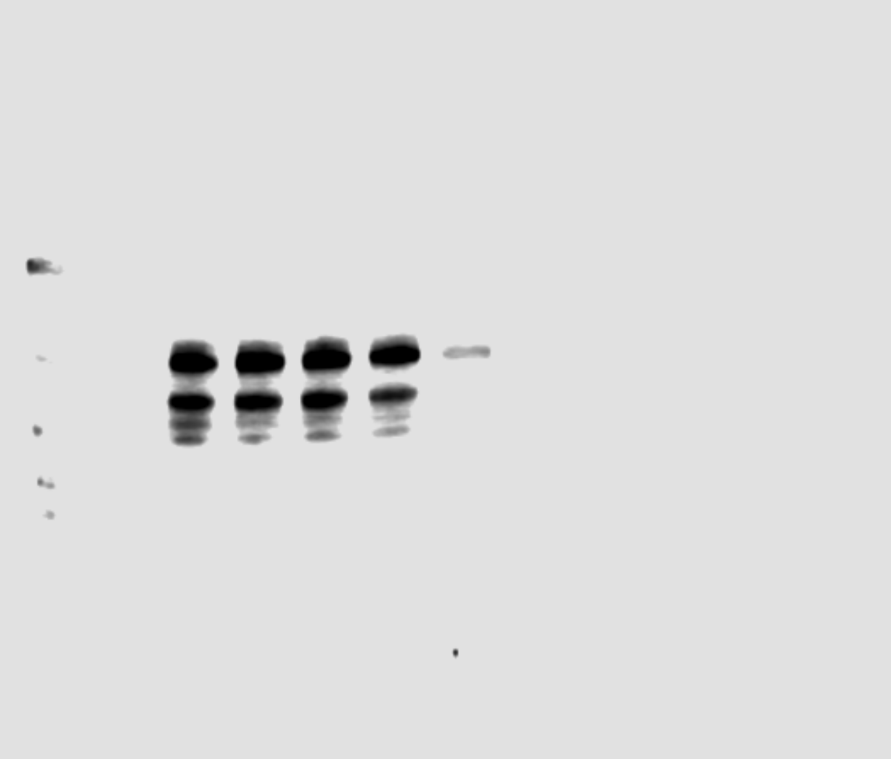


3CD

73 kDa

53kDa

3D

**Supplementary Figure 3:** Full blot detection of Actin. Actin was stripped and blot on the same blot with VP0 and VP2 detection. Stripping was not completely done, residues of VP0 and VP2 detection remains on the blot.


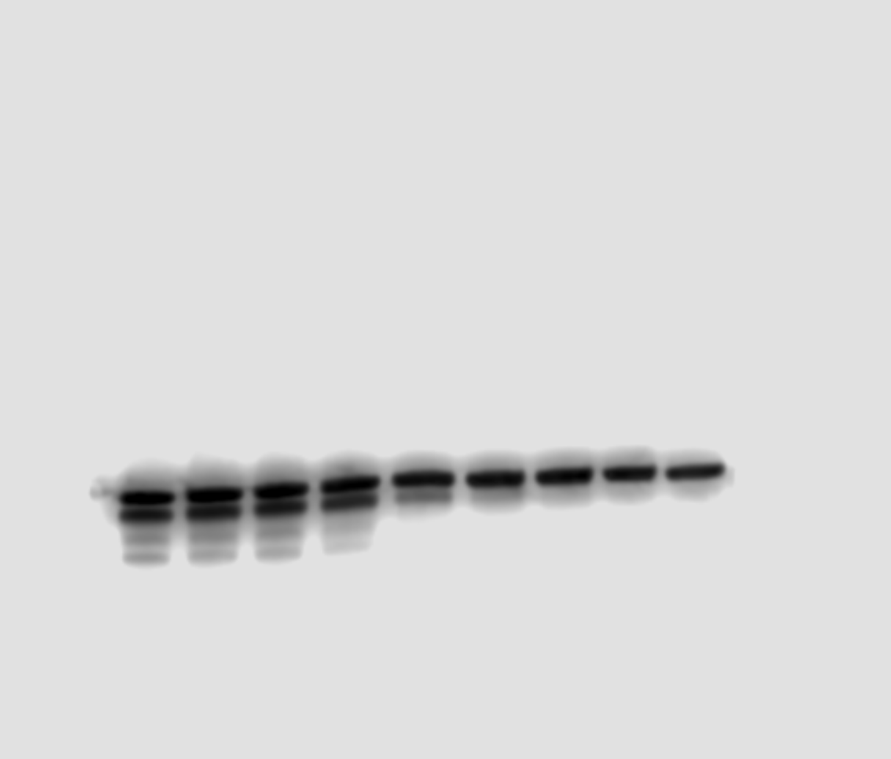


VP2

VP0

Actin

28 kDa

42 kDa

36 kDa
